# Supplementary material for: Using apelin-based synthetic Notch receptors to detect angiogenesis and treat solid tumors
Source: Nat Commun. 2020 May 1;11:2163. doi: 10.1038/s41467-020-15729-4 (PMC7195494; doi:10.1038/s41467-020-15729-4)
Supplement: Supplementary file 1 — Supplementary Information [file 41467_2020_15729_MOESM1_ESM.pdf]

Supplementary Information PDF

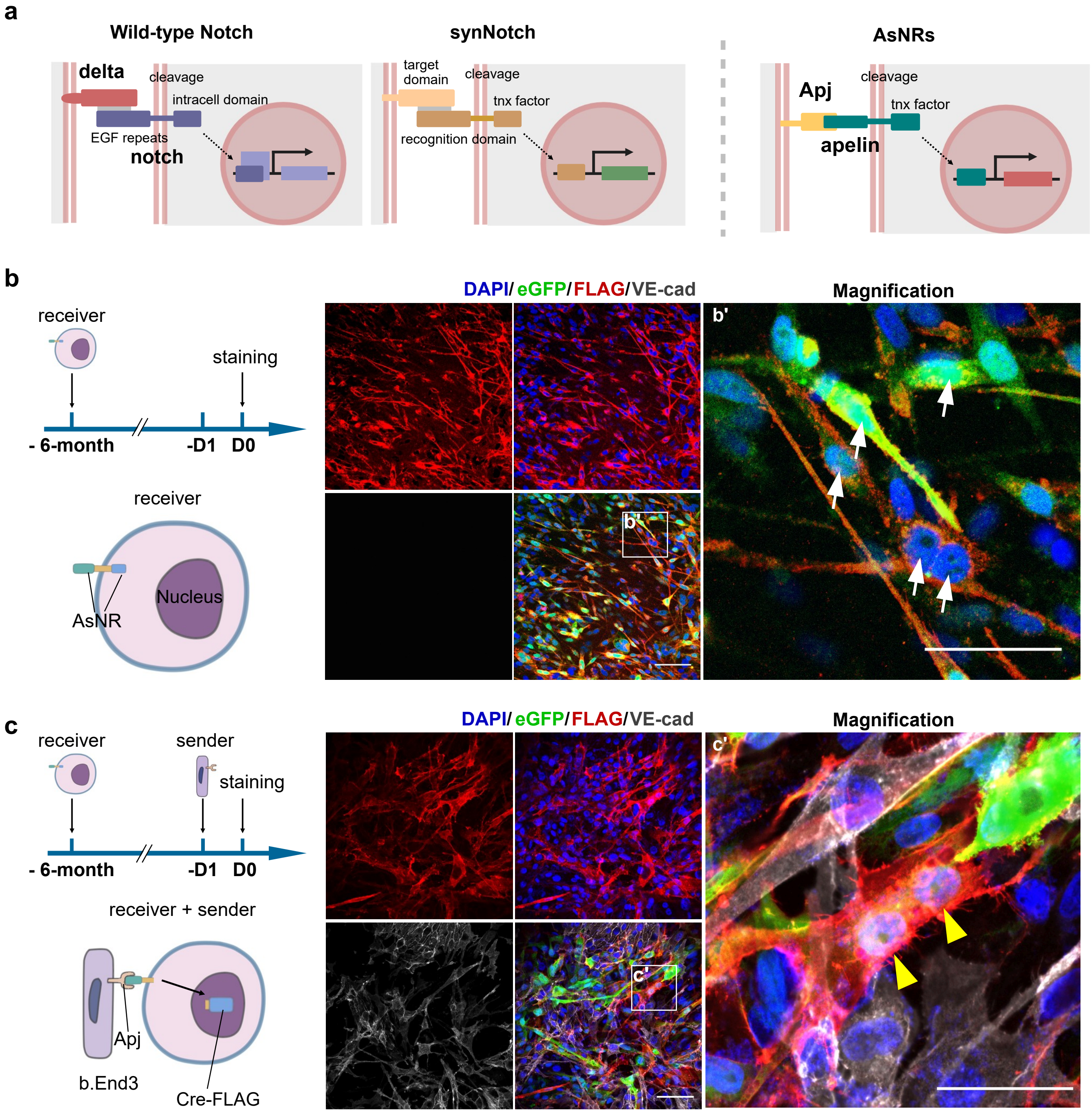

Supplementary figure 1. Engineered cells with AsNRs can sense the Apj+ endothelial cells. Related to Figure 1.

a. Schematic showing the AsNRs system. Wild-type Notch: the EGF repeats that binds to Delta of the partner cell, and the intracellular domain released by ligand-induced cleavage. synNotch: both the extracellular and intracellular domains can be flexibly replaced. AsNRs: the extracellular domain is replaced by modified apelin to specifically sense Apj+ cells. **b.** Schematic on the left showed the strategy of experiments. Immunostaining for GFP, FLAG and VE-cad showing that intracellular domains were still on the membrane after 6-month culture. **c.** Intracellular domains entered nucleus after cell-cell contact, indicating that cells customized with AsNRs keep the capability to sense the endothelial cells in long-term cultivation. Scale bars = 100μm in **b** and **c**; 50μm in **b**' and **c**'.

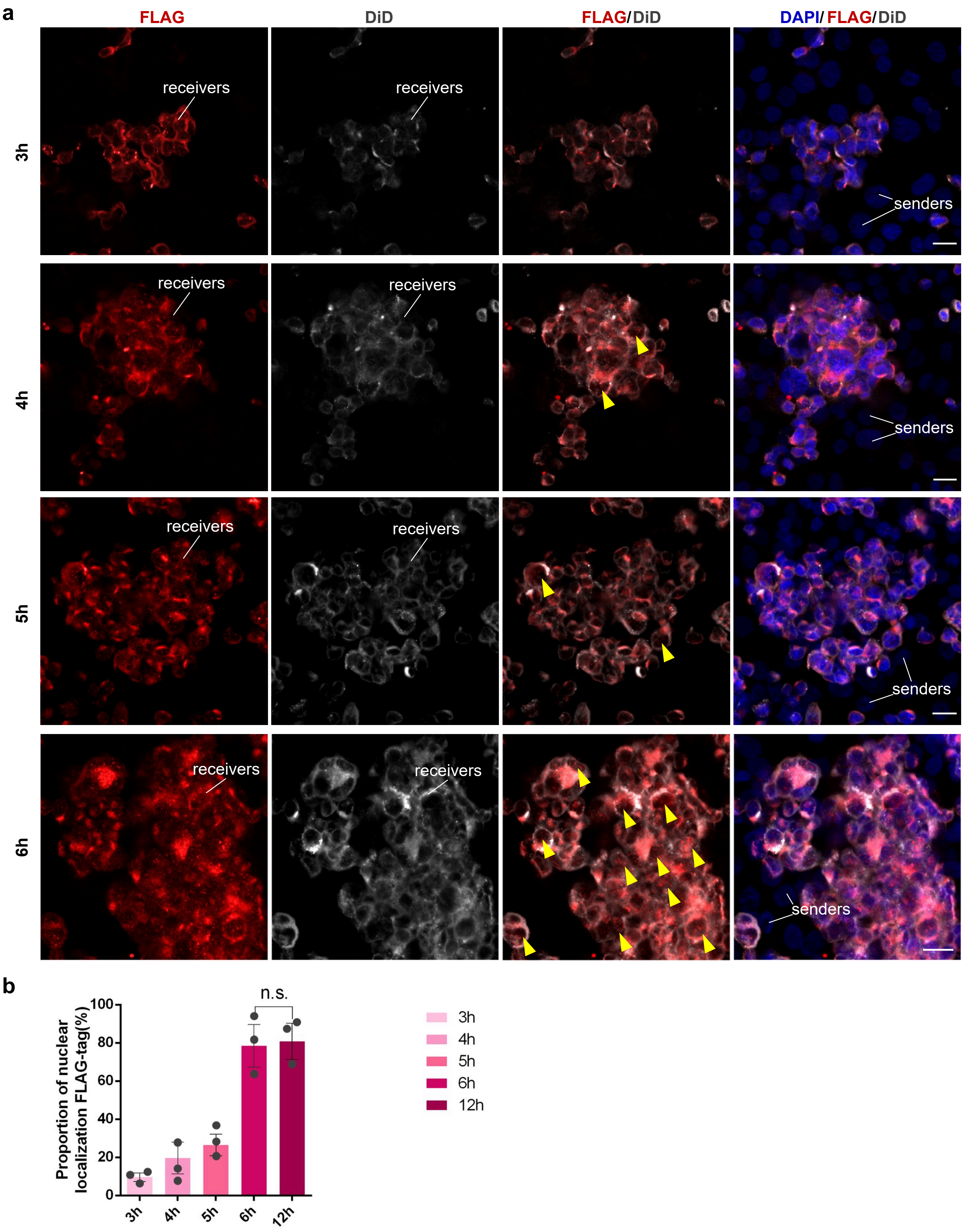

Supplementary figure 2. The intracellular domains will be released rapidly from membrane at 6-hour after sensing sender cells. Related to Figure 1.

a. Immunostaining for GFP, FLAG and VE-cad showing the process that intracellular domains were released from membrane(yellow arrowheads) between 2-hour and 6-hour after delivering sender cells. **b.** Quantification of proportion of nuclear localization FLAG-tag between 2-hour and 12-hour, indicating they were released rapidly from membrane at 6-hour after sensing sender cells.(n=3 wells per group). Scale bars = 20μm in **a**. Error bars: SEM.

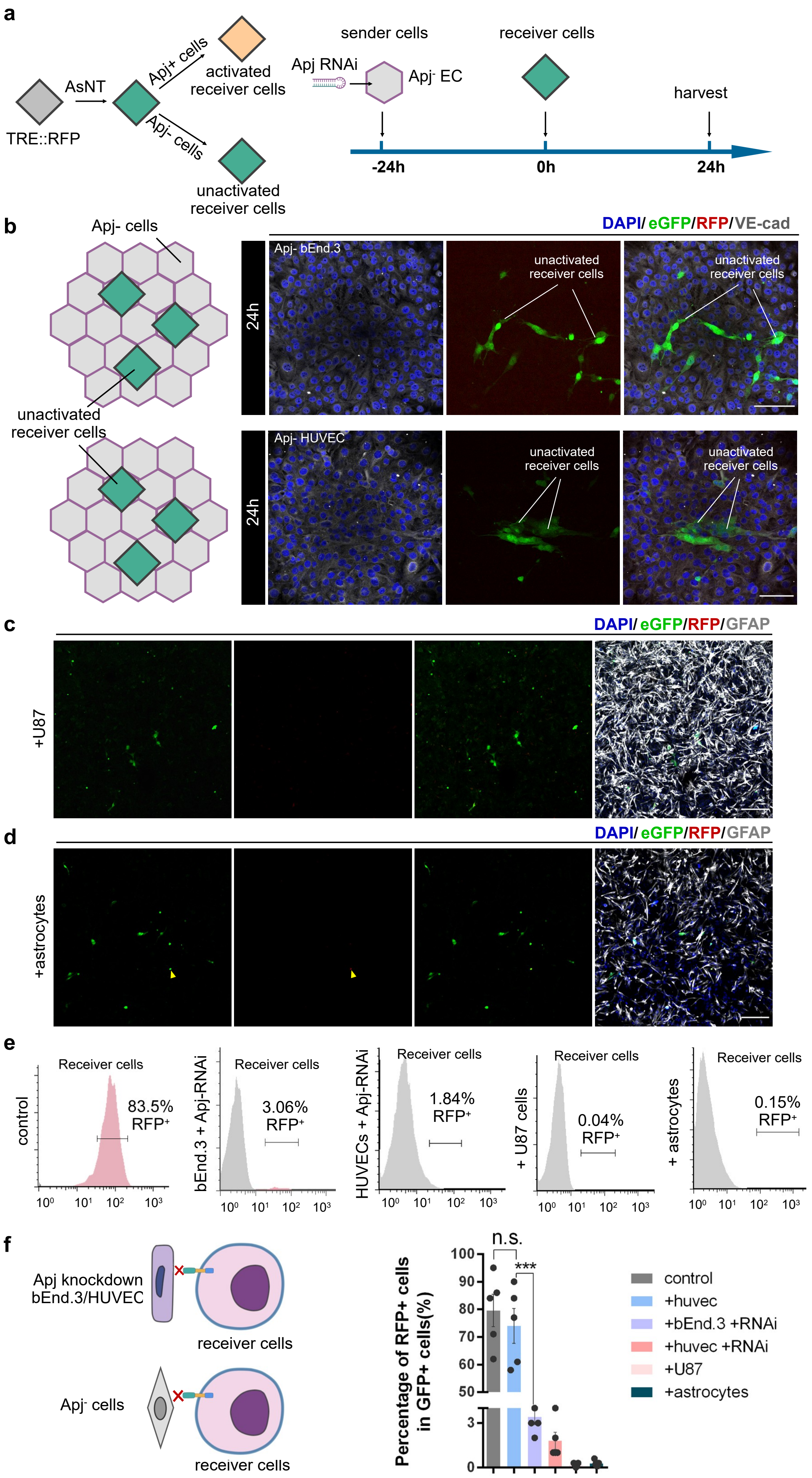
a
sender cells
receiver cells
Apj RNAi
Apj- EC
AsNT
TRE::RFP
Apj+ cells
Apj- cells
activated receiver cells
unactivated receiver cells
harvest
-24h
0h
24h
b
Apj- cells
unactivated receiver cells
DAPI/ eGFP/ RFP/ VE-cad
24h
Apj- bEnd.3
Apj- HUVEC
c
DAPI/ eGFP/ RFP/ GFAP
+U87
d
+astrocytes
e
Receiver cells
control
83.5% RFP+
bEnd.3 + Apj-RNAi
3.06% RFP+
HUVECs + Apj-RNAi
1.84% RFP+
+ U87 cells
0.04% RFP+
+ astrocytes
0.15% RFP+
f
Apj knockdown bEnd.3/HUVEC
receiver cells
Apj- cells
Percentage of RFP+ cells in GFP+ cells(%)
n.s.
***
control
+huvec
+bEnd.3 +RNAi
+huvec +RNAi
+U87
+astrocytes

Supplementary figure 3. AsNRs can merely sense Apj and be exclusively induced by proliferating endothelial cells. Related to Figure 2.
a. Experimental strategy to knockdown Apj in bEnd.3/HUVEC and co-culture with receiver cells. **b.** Immunostaining for VE-cad showing that RFP reporter were not activated when the Apj of endothelial cells (bEnd.3 and HUVEC) was knockdown. **c.** Immunostaining for GFAP showing that RFP reporter was not activated when receiver cells were co-cultured with U87 cell line. **d.** Immunostaining for GFAP showing that quite few receiver cells turned red (yellow arrowhead) when receiver cells were co-cultured with primary astrocytes. **e** and **f.** Quantitative analysis by FACS showing that engineering cells with AsNRs merely cognized Apj+ cells; control: HUVECs with non-relevant RNAi . Scale bars = 100μm in **b**; 200μm in **c** and **d**. Error bars: SEM. Significance determined by Students t-test: n.s. $p>0.05$; *** $p<0.001$ (n=5 wells in **f**).

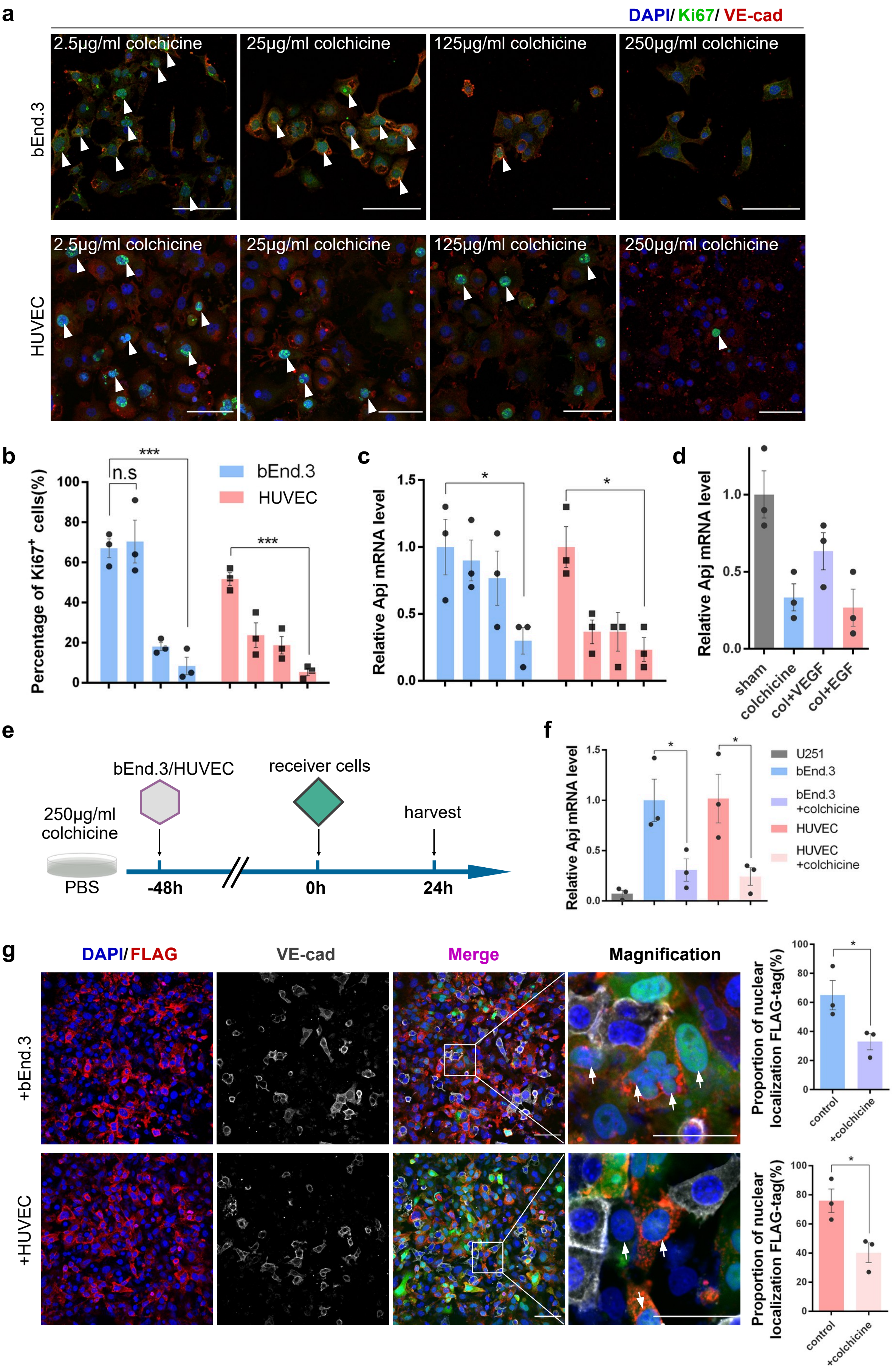
a
DAPI/ Ki67/ VE-cad
2.5μg/ml colchicine
25μg/ml colchicine
125μg/ml colchicine
250μg/ml colchicine
bEnd.3
HUVEC
b
Percentage of Ki67+ cells(%)
n.s
***
bEnd.3
HUVEC
c
Relative Apj mRNA level
*
d
Relative Apj mRNA level
sham
colchicine
col+VEGF
col+EGF
e
250μg/ml colchicine
PBS
bEnd.3/HUVEC
receiver cells
harvest
-48h
0h
24h
f
Relative Apj mRNA level
U251
bEnd.3
bEnd.3 +colchicine
HUVEC
HUVEC +colchicine
g
DAPI/FLAG
VE-cad
Merge
Magnification
+bEnd.3
+HUVEC
Proportion of nuclear localization FLAG-tag(%)
control
+colchicine

Supplementary figure 4. AsNRs can merely sense Apj and be exclusively induced by proliferating endothelial cells. Related to Figure 2.

a. Immunostaining for Ki67 (a marker of proliferation) and VE-cad showing that the number of Ki67+ cells (white arrowheads) decreased after adding colchicine. **b.** Quantitative analysis indicating that 250μg/ml colchicine significantly inhibited the proliferation of bEnd.3/HUVEC. (n=3 wells per group) **c.** qPCR analysis showing that the mRNA level of Apj and Ki67 expression showed a similar downward trend.(n=3 wells per group) **d.** Apj expression was rescued in high VEGF condition.(n=3 wells per group) **e.** Experimental strategy to inhibit proliferation of endothelial cells using colchicine. See also supplementary figure 3. **f.** Analyzed expression of Apj in bEnd.3 and HUVEC by qPCR after proliferation of bEnd.3 and HUVEC was inhibited.(n=3 wells per group) **g.** The percentage of nuclear localization FLAG-tags decreased when the proliferation of senders was inhibited, comparing with normal senders. Boxed regions are magnified in the right panel.(n=3 wells per group). Scale bars = 100μm in **a** and **g**. Error bars: SEM. Significance determined by Students t-test: * $p<0.05$, ** $p<0.01$, *** $p<0.001$.

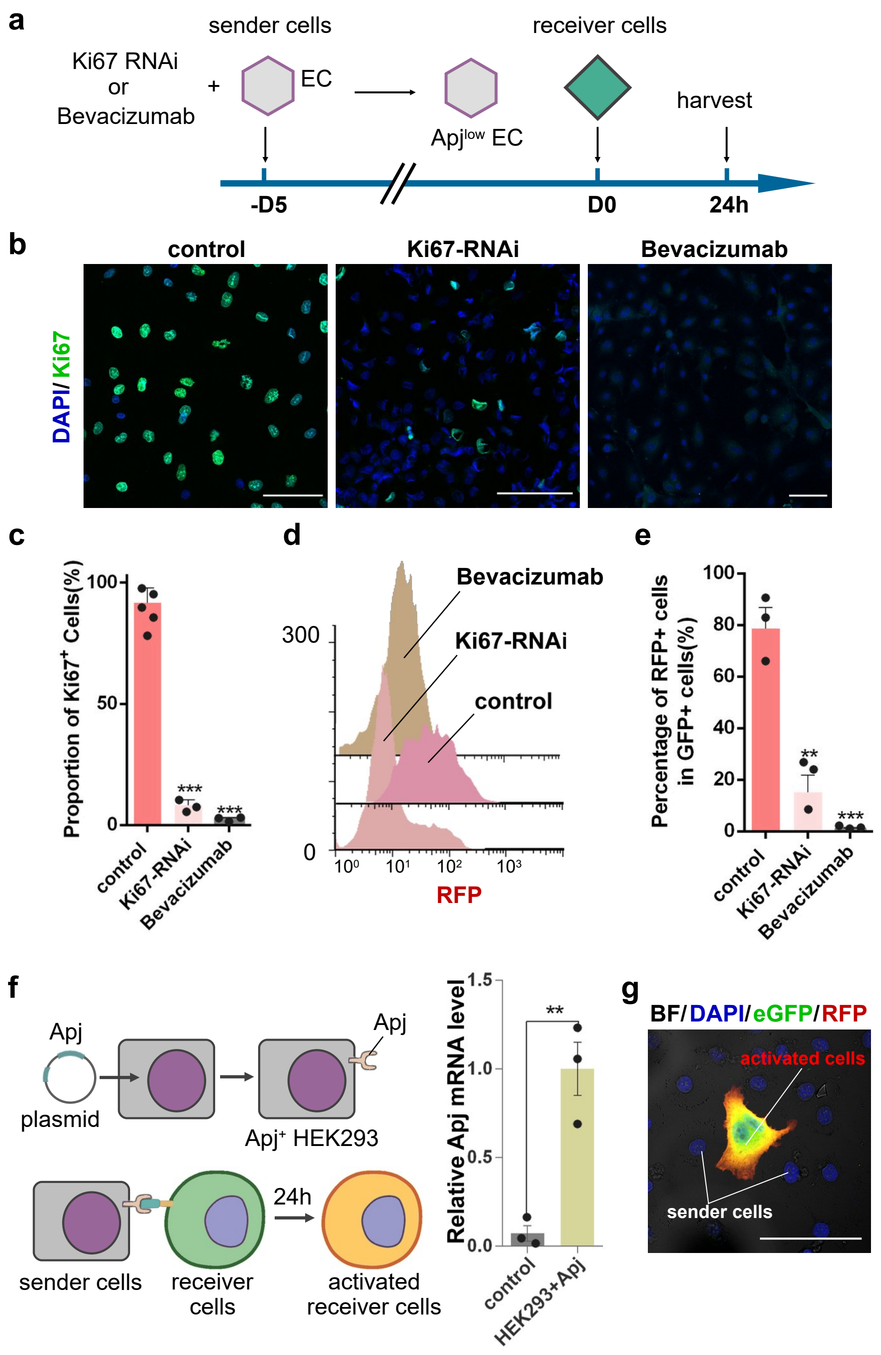

Supplementary figure 5. AsNRs can merely sense Apj and be exclusively induced by proliferating endothelial cells. Related to Figure 2.
a. Experimental strategy to inhibit the proliferation of HUVEC and then co-culture with receiver cells. **b** and **c.** Immunostaining for Ki67 showing that the proliferation of HUVEC was inhibited. (control: n=5; RNAi and bevacizumab: n=3 per group) **d** and **e.** Quantitative analysis by FACS shows that RFP reporter were not activated when the proliferation of HUVEC was inhibited.(n=3 samples per group) **f.** Transfecting cell line HEK293 to express Apj and test the Apj mRNA by qPCR.(n=3 wells per group) **g.** Imaging of fluorescence shows that Apj+ HEK293 cell line can activate the engineered cells with AsNRs. col: colchicine. BF: bright-field. Scale bars = 100μm in **b**; 50μm in **g.** Error bars: SEM. Significance determined by Students t-test: *** $p<0.001$, ** $p<0.01$, * $p<0.05$.

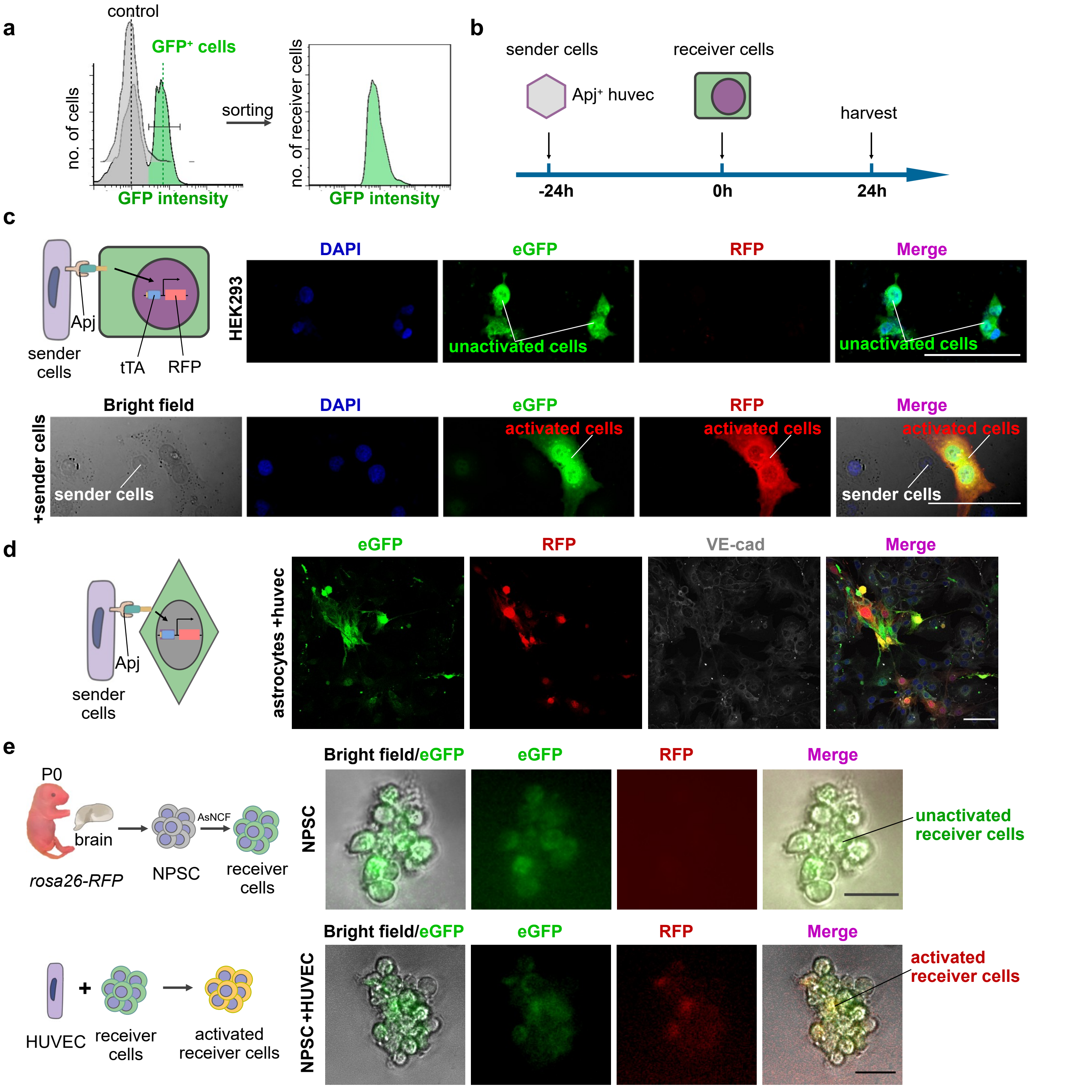

Supplementary figure 6. AsNRs can drive the customized programs in multiple cells. Related to Figure 3.

a. HEK293 cells with TRE::RFP were customized with AsNRs (intracellular domain: tTA), and sorted by FACS. **b.** Schematic showing the strategy of experiments. All samples were harvested on 24-hour after sending HUVEC cell line to the receiver cells. **c.** Fluorescence images showed that AsNRs can drive the customized programs in HEK293 cell line. **d.** Primary astrocytes with TRE::RFP were customized with AsNRs, and these primary cells turned red after sensing the HUVEC. **e.** The Schematic on the left showed the experimental processes. The efficiency of AsNC (intracellular domain: cre recombinases) was tested in neural progenitor/stem cells. The fluorescence images indicated that a few cells turned red after contacting huvec (huvec were removed). Scale bars = 50μm.

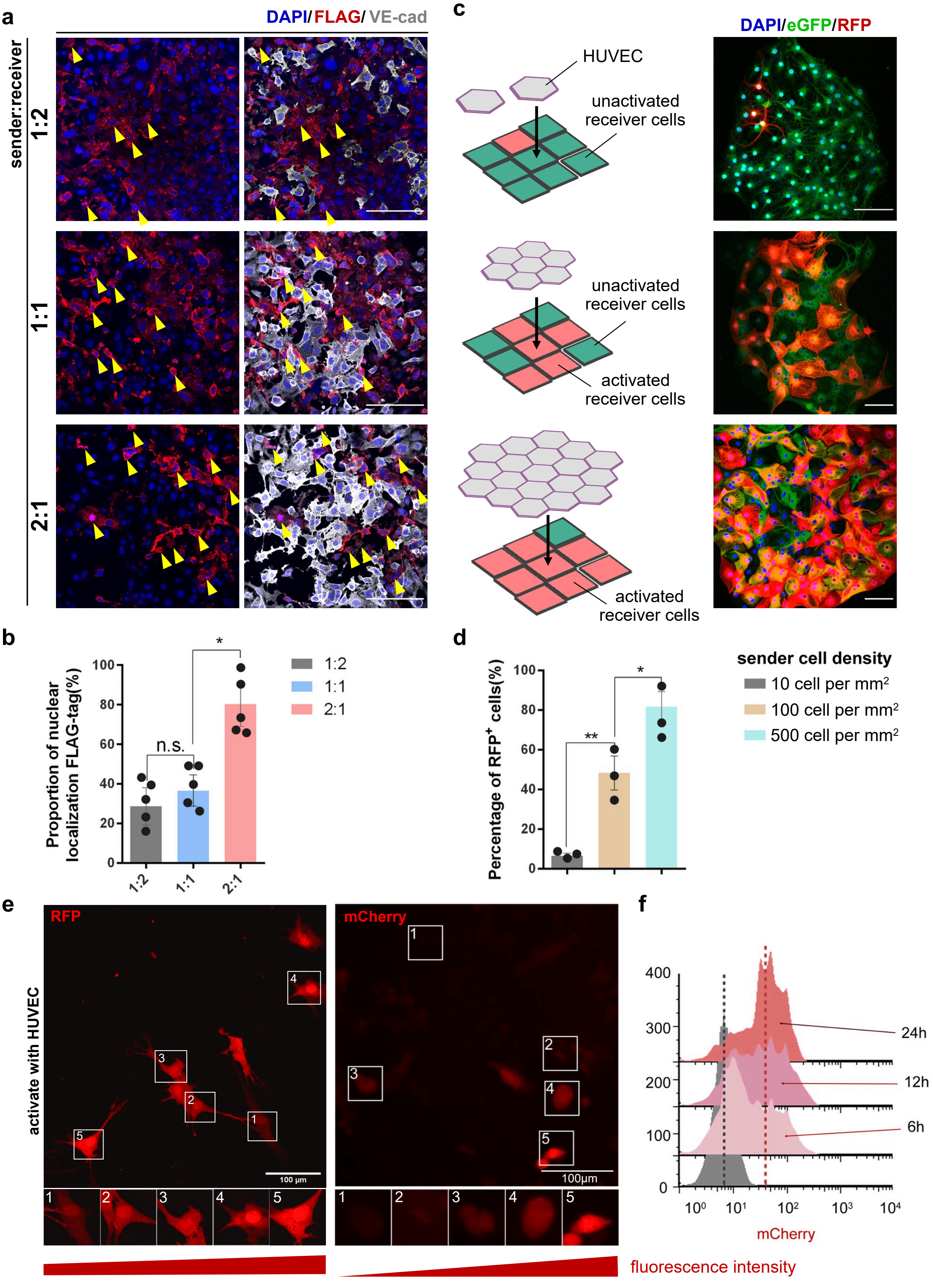

Supplementary figure 7. The proportion of activation increases as the number of sender cells increases.

a. Immunostaining for FLAG and VE-cad showing that receiver cells of nuclear localized FLAG-tags (yellow arrowheads) were biased towards HUVEC cells. **b.** Quantitative analysis shows the proportion of nuclear localized FLAG-tags significantly increased at the ratio of sender: receiver is 2:1. **c** and **d.** Intensive sender cells (HUVEC) lead to higher proportion of receiver cells turned red, compared with sparse number of sender cells (HUVEC cells were removed). **e** and **f.** Fluorescence images and FACS showing that RFP is a more sensitive reporter compared with mCherry in synNotch system. Scale bars = 100μm. Error bars: SEM. Significance determined by Students t-test: ** $p<0.01$, * $p<0.05$ (n=5 wells per group in **b**, and n=3 wells per group in **d**).

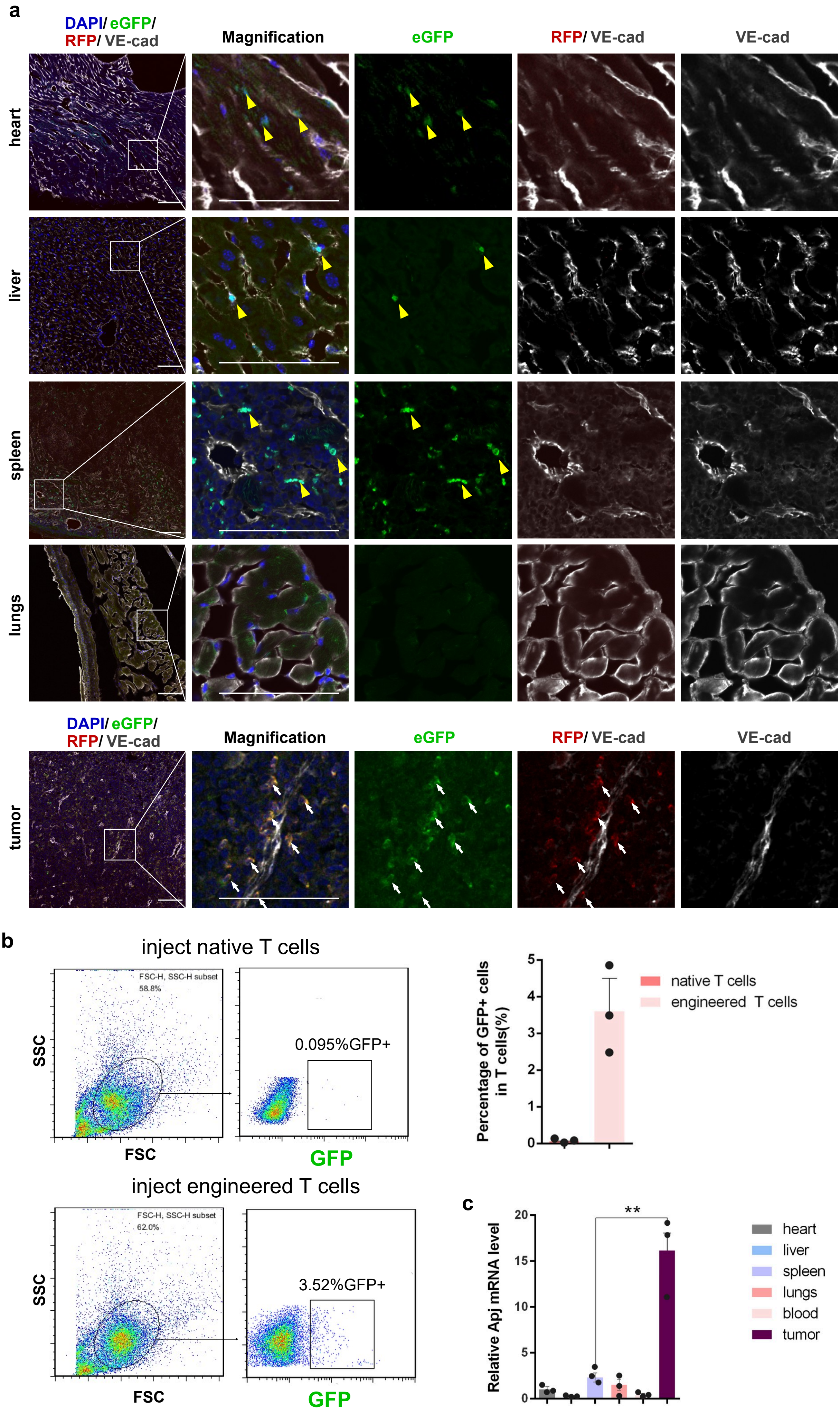

a
DAPI/ eGFP/ RFP/ VE-cad
Magnification
eGFP
RFP/ VE-cad
VE-cad
heart
liver
spleen
lungs
tumor
b
inject native T cells
FSC-H, SSC-H subset
58.8%
SSC
FSC
0.095%GFP+
GFP
inject engineered T cells
FSC-H, SSC-H subset
62.0%
3.52%GFP+
Percentage of GFP+ cells in T cells(%)
native T cells
engineered T cells
c
Relative Apj mRNA level
**
heart
liver
spleen
lungs
blood
tumor

Supplementary figure 8. Engineered T cells can be delivered throughout the body, but they can be only activated in tumors. Related to Figure 5 and 6.

a.Immunostaining for VE-cad on heart, liver, spleen, lung and tumor sections showing that GFP+ cells in or around blood vessels, but they were only activated in tumors. **b.** Quantification of GFP+ cells in T cells of blood, indicating that there are few GFP+ cells in blood. **c.** qPCR for Apj in various tissues showing the mRNA level of Apj was significantly increased in tumor, indicating that AsNRs are specific for Apj. Scale bars = 100μm in **a**. Error bars: SEM. Significance determined by Students t-test: ** $p<0.01$ (n=3 mice per group).

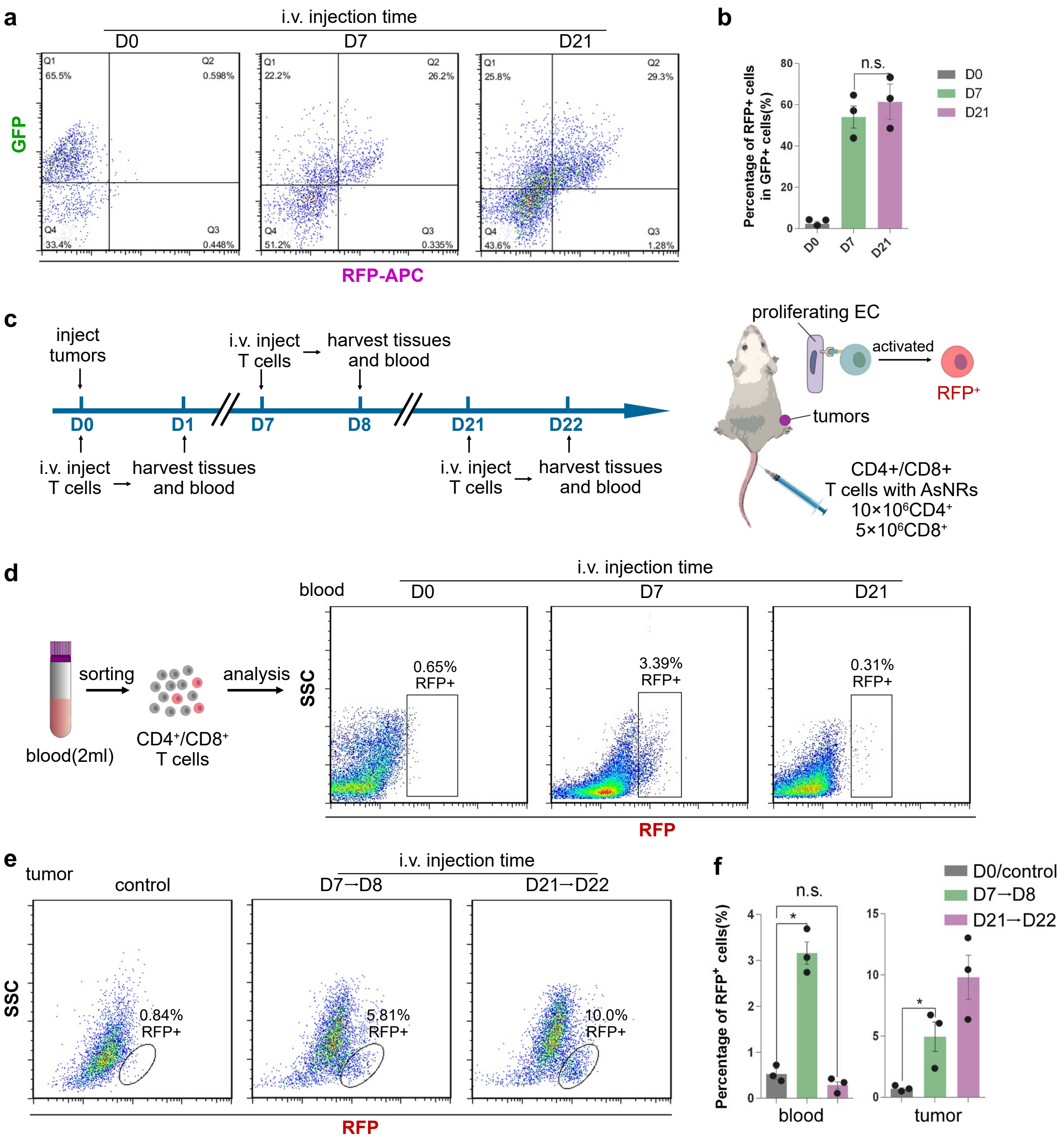

Supplementary figure 9. Engineered cells with AsNRs can sense the sprouting vessels of tumors in adults. Related to Figure 5 and 6.

a and b. Quantitivative analysis of proportion of turned red cells in GFP⁺ cells in vivo. **c.** Schematic showing the strategy of experiments. Engineered T cells were i.v. injected on 24-hour before harvesting tumors and blood. The engineered cells would turn red if they can sense the endothelium of sprouting vessels. **d.** CD4+ and CD8+ T cells were sorted from blood, and the proportion of RFP-positive cells increased significantly at D7, compared with D0 and D21. **e and f.** In glioma models, the proportion of RFP⁺ T cells increased at D21, which indicated that engineered cells was retargeted to tumors as tumors growth. Error bars: SEM. Significance: * $p<0.05$ (n=3 samples per group). Each blood sample comes from two mice.

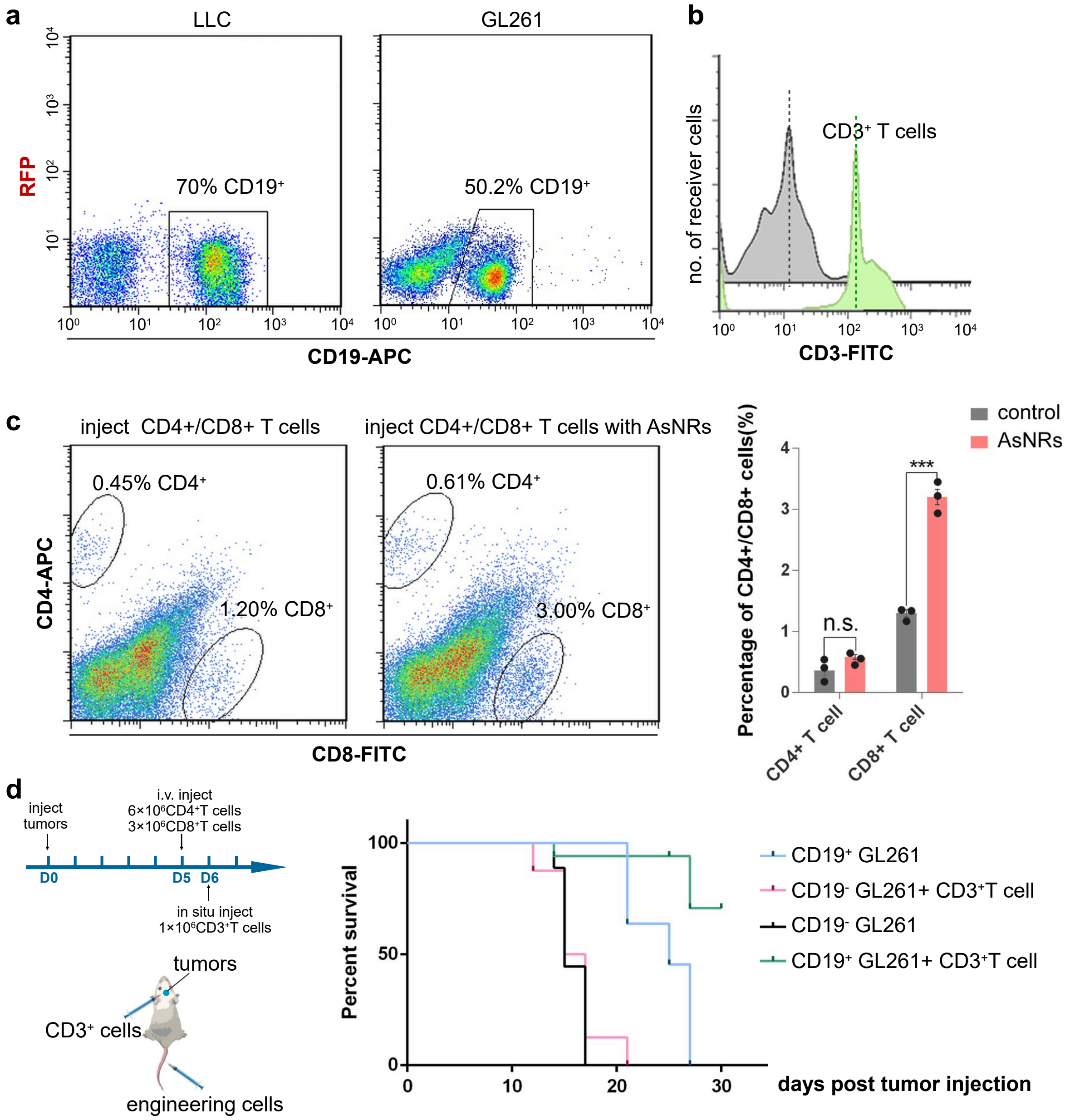

Supplementary figure 10. CD4+ and CD8+ T cells customized with AsNRs can inhibit solid tumors Related to Figure 7.

a. Sorting CD19+ LLC cells to generate CD19+ tumor models. **b.** Quantitative analysis of the proportion of CD3+ T cells after sorting by microbeads. **c.** Quantitation of CD4+ and CD8+ T cells in tumor indicated that the proportion of CD8+ T cells increased after injecting engineered T cells. **d.** Survival analysis showing that survival percentage of glioma-bearing mice increased after treating with engineering T cells. Error bars: SEM. Significance determined by Students t-test: *** $p<0.001$, n.s. $p>0.05$ (n=5 mice per group in **c**; n=9 mice per group in **d**).

Supplementary Table 1. Key sequence-based reagents.

synNotch core (peptide sequence from human notch1 – uniprot Q01705)<br>Apelin--<br>PCVGSNPCYNQGTCEPTSENPFYRCLCPAKFNGLLCHILDYSFTGGAGRDIPPPQIEEAC<br>ELPECQVDAGNKVCNLQCNNHACGWDGGDCSLNFNDPWKNCTQSLQCWKYFSDGH<br>CDSQCNSAGCLFDGFDCQLTEGQCNPLYDQYCKDHFSDGHCDQGCNSAECEWDGLD<br>CAEHVPERLAAGTLVLVVLLPPDQLRNNSFHFLRELSHVLHTNVVFKRDAQGQQMIFP<br>YYGHEEELRKHPIKRSTVGWATSSLLPGTSGGRQRRELDPMDIRGSIVYLEIDNRQCVQ<br>SSSQCFQSATDVAAFLGALASLGSLNIPYKIEAVKSEPVEPPLPSQLHLMYVAAAAFVL<br>LFFVGCGVLLSRKRRRQLCIQKL—tTA/cre
Anti-Apj sequence (peptide sequence from human apelin)<br>MNLRLCVQALLLLWLSLTAVCGVPLMLPPDGTGLEEGSMRYLVKPRTSRTGPGAWQ<br>GGRRKFRRQRPRLSHKGPMPF

Supplementary Table 2:

Figure	Comparison	P value
Fig.1 e	U251 vs. b.End3	0.0042
	U251 vs. HUVEC	0.0023
Fig.1 f	HUVEC vs. b.End3	0.296
	HUVEC vs. b.End3	0.226
Fig.2 b	b.End3 vs. b.End3+RNAi	0.0056
	HUVEC vs. HUVEC+RNAi	0.015
Fig.2 d	b.End3: contact vs. no contact	0.0004
	HUVEC: contact vs. no contact	0.0003
Fig.2 f	b.End3 vs. b.End3+RNAi	0.0062
	HUVEC vs. HUVEC+RNAi	0.0352
Fig.2 g	b.End3 vs. b.End3+RNAi	0.0225
	HUVEC vs. HUVEC+RNAi	0.0072
Fig.3 e	12h vs. 24h	0.017
Fig.4 e	U251 vs. HUVEC	0.0001
Fig.5 e	Blood: D0 vs. D7-8	0.013
	Tumor D7-8 vs. D21-22	0.008
Fig.5 b	Control vs. 13-week	0.0008
Fig.5 e	Control vs. 13-week	0.0001
Fig.7 c	With HUVEC: NTC vs. ETC	0.004
	NTC with HUVEC vs. ETC without HUVEC	0.149
Fig.7 c	With HUVEC: NTC vs. ETC	0.004
Supplementary fig.2b	6h vs. 12h	
Supplementary fig.3f	HUVEC vs. RNAi	0.0001
	Control vs. HUVEC	0.5321
Supplementary fig.4b	b.End3: 2.5μg vs. 250μg	0.0008
	HUVEC: 2.5μg vs. 250μg	0.0002
Supplementary fig.4f	b.End3 vs. b.End3+col	0.0387
	HUVEC vs. HUVEC+col	0.0122

Supplementary fig.4g	b.End3: control vs. col	0.0370
	HUVEC: control vs. col	0.0277
Supplementary fig.5e	control vs. Ki67-RNAi	0.0040
	control vs. bevacizumab	0.0007
Supplementary fig.5f	control vs. Apj^+ HEK293	0.004
Supplementary fig.7b	1:1 vs. 2:1	0.032
	1:1 vs. 1:2	0.538
Supplementary fig.7d	10 cell/mm^2 vs. 100 cell/mm^2	0.0437
	100 cell/mm^2 vs. 500 cell/mm^2	0.0087
Supplementary fig.8c	Tumor vs. spleen	0.0018
Supplementary fig.9b	D7 vs. D21	0.435
Supplementary fig.9f	Blood: D0 vs. D7-8	0.020
	Tumor D7-8 vs. D21-22	0.024
Supplementary fig.10c	CD4: Control vs. AsNRs	0.5230
	CD8: Control vs. AsNRs	0.0002
