# Supplementary material for: Using apelin-based synthetic Notch receptors to detect angiogenesis and treat solid tumors
Source: Nat Commun. 2020 May 1;11:2163. doi: 10.1038/s41467-020-15729-4 (PMC7195494; doi:10.1038/s41467-020-15729-4)
Supplement: Supplementary file 3 — Reporting Summary [file 41467_2020_15729_MOESM3_ESM.pdf]

nature research

Corresponding author(s): Zhu Jianhong DBPR

Last updated by author(s): Feb, 19, 2020

Reporting Summary

Nature Research wishes to improve the reproducibility of the work that we publish. This form provides structure for consistency and transparency in reporting. For further information on Nature Research policies, see Authors & Referees and the Editorial Policy Checklist .

Statistics

For all statistical analyses, confirm that the following items are present in the figure legend, table legend, main text, or Methods section.

n/a	Confirmed	
☐	☒	The exact sample size (*n*) for each experimental group/condition, given as a discrete number and unit of measurement
☐	☒	A statement on whether measurements were taken from distinct samples or whether the same sample was measured repeatedly
☐	☒	The statistical test(s) used AND whether they are one- or two-sided<br>*Only common tests should be described solely by name; describe more complex techniques in the Methods section.*
☐	☒	A description of all covariates tested
☐	☒	A description of any assumptions or corrections, such as tests of normality and adjustment for multiple comparisons
☐	☒	A full description of the statistical parameters including central tendency (e.g. means) or other basic estimates (e.g. regression coefficient) AND variation (e.g. standard deviation) or associated estimates of uncertainty (e.g. confidence intervals)
☐	☒	For null hypothesis testing, the test statistic (e.g. *F*, *t*, *r*) with confidence intervals, effect sizes, degrees of freedom and *P* value noted<br>*Give P values as exact values whenever suitable.*
☒	☐	For Bayesian analysis, information on the choice of priors and Markov chain Monte Carlo settings
☐	☒	For hierarchical and complex designs, identification of the appropriate level for tests and full reporting of outcomes
☒	☐	Estimates of effect sizes (e.g. Cohen's *d*, Pearson's *r*), indicating how they were calculated

Our web collection on statistics for biologists contains articles on many of the points above.

Software and code

Policy information about availability of computer code

Data collection	NIS-Elements(AR 4.60.00; Nikon); BD CellQuest Pro (ver5.2; BD)
Data analysis	ImageJ(FIJI win-64;NIH); Prism 6 (graphpad); SPSS software (ver. 13.0; SPSS Inc.); FlowJo (ver.7.6;TreeStar)

For manuscripts utilizing custom algorithms or software that are central to the research but not yet described in published literature, software must be made available to editors/reviewers. We strongly encourage code deposition in a community repository (e.g. GitHub). See the Nature Research guidelines for submitting code & software for further information.

Data

Policy information about availability of data

All manuscripts must include a data availability statement. This statement should provide the following information, where applicable:

- Accession codes, unique identifiers, or web links for publicly available datasets
- A list of figures that have associated raw data
- A description of any restrictions on data availability

The source data has been deposited in the Dryad Digital Repository (https://doi.org/10.5061/dryad.9ghx3ffdm). The data that support the findings of this study are available from the corresponding author upon reasonable request.

Field-specific reporting

Please select the one below that is the best fit for your research. If you are not sure, read the appropriate sections before making your selection.

☒ Life sciences ☐ Behavioural & social sciences ☐ Ecological, evolutionary & environmental sciences

For a reference copy of the document with all sections, see nature.com/documents/nr-reporting-summary-flat.pdf

Life sciences study design

All studies must disclose on these points even when the disclosure is negative.

[illegible]ample size	3-5 samples for immunofluorescence or FACS per gourp. 5-8 samples for quantification per group. No statistical method. All attempts at replication were successful.
Data exclusions	No data were excluded
[illegible]eplication	The animal experiments were replicated five times at least. Cell culture and transfection were replicated three times at least. All attempts at replication were successful.
Randomization	All samples were allocated randomly
Blinding	The investigators were blinded to group allocation during data collection and analysis.

Reporting for specific materials, systems and methods

We require information from authors about some types of materials, experimental systems and methods used in many studies. Here, indicate whether each material, system or method listed is relevant to your study. If you are not sure if a list item applies to your research, read the appropriate section before selecting a response.

Materials & experimental systems

n/a	Involved in the study	
☐	☒	Antibodies
☐	☒	Eukaryotic cell lines
☒	☐	Palaeontology
☐	☒	Animals and other organisms
☒	☐	Human research participants
☒	☐	Clinical data

Methods

n/a	Involved in the study	
☒	☐	ChIP-seq
☐	☒	Flow cytometry
☒	☐	MRI-based neuroimaging

Antibodies

[illegible]ntibodies used

Rabbit anti-FLAG 1:200 Abcam Cat# ab205606; Goat anti-Mouse-GFP (FITC conjugate) 1:200 Abcam Cat# ab6662; Goat anti-VE-cad 1:100 R&D System Cat#AF1002; Rb anti-GFAP 1:500 Abcam Cat#ab7260; Chicken anti-GFAP 1:800 Abcam Cat#ab4674 ;Rb anti-RFP 1:1000 Rockland Cat#600-401-379; Rat anti-RFP 1:200 ChromoTek Cat#ABIN334653; Rb anti-Ki67 1:200 Abcam Cat#ab15580; Donkey Anti-Rabbit IgG H&L (Alexa Fluor® 488) 1:1000 Abcam Cat#ab150073 Donkey Anti-Goat IgG H&L (Alexa Fluor® 488) 1:1000 Abcam Cat#ab150129 Goat Anti-Chicken IgY H&L (Alexa Fluor® 488) 1:1000 Abcam Cat#ab150169 Donkey Anti-Mouse IgG H&L (Alexa Fluor® 488) 1:1000 Abcam Cat#ab150105 Donkey Anti-Rabbit IgG H&L (Alexa Fluor® 555) 1:1000 Abcam Cat#ab150074 Donkey Anti-Rat IgG H&L (Alexa Fluor® 555) 1:1000 Abcam Cat#ab150154 Mouse Anti-CD3/CD28 1:3000 Life Technologies Cat#11456D; Donkey Anti-Rabbit IgG H&L (Alexa Fluor® 647) 1:1000 Abcam Cat#ab150075
Rat anti-CD4-APC 1:100 Biolegend Cat#100515
Rat anti-CD8-APC 1:100 Biolegend Cat#100712
Rat anti-CD8-FITC 1:100 Biolegend Cat#100706
Hamster anti-CD69-APC 1:100 Biolegend Cat#104513
Rat anti-CD3-FITC 1:100 Biolegend Cat#100203
APC anti-CD19 1:100 Biolegend Cat#115512

[illegible]alidation

Goat anti-VE-cad: VE-cad is a marker of the endothelial cells, and the pattern of the immunostaining on brain sections is consistent with the pattern of vessels.
Rb anti-GFAP and Chicken anti-GFAP : GFAP is a marker of astrocytes/NSCs, and the pattern of the immunostaining on hippocampus sections is consistent with the pattern of astrocytes/NSCs.
Rb anti-Ki67: Ki67 is a marker of proliferating cells, the immunostaining on neurons and NSCs showing a correct pattern.
Rb anti-FLAG: immunostaining on cells infected with FLAG DNA showing positive signal, but negative in control cells.
APC anti-mouse CD4, APC anti-mouse CD8 and FITC anti-mouse CD8: CD4 and CD8 are two markers of CD4+ T cells and CD8+ T cells respectively, and data of FACS showed that CD4 and CD8 can label CD4+ Tcells and CD8+ T cells respectively.

Eukaryotic cell lines

Policy information about cell lines

Cell line source(s)	HEK293 Human embryonic kidney 293 cells (The cell bank of Shanghai Institutes of Biological Sciences), HEK293T (The cell bank of Shanghai Institutes of Biological Sciences), LLC: Lewis lung carcinoma cells (The cell bank of Shanghai Institutes of Biological Sciences), GL261: murine glioma cell line (Huashan Hospital), U251: U-251 MG (Huashan Hospital), U87: human primary glioblastoma cell line (Huashan Hospital), bEnd.3: mouse brain cell line (The cell bank of Shanghai Institutes of Biological Sciences), HUVEC: Human umbilical vein endothelial cells (The cell bank of Shanghai Institutes of Biological Sciences)
Authentication	DNA was extracted with Axygen's Genome Extraction Kit, amplified using the 21-STR amplification protocol, and the STR loci were detected on the ABI 3730XL.
Mycoplasma contamination	all cell lines negative for mycoplasma
Commonly misidentified lines (See ICLAC register)	no commonly misidentified lines used in the study

Animals and other organisms

Policy information about studies involving animals; ARRIVE guidelines recommended for reporting animal research

Laboratory animals	The mice (male, 10-14 weeks old) used in the experiment are all from the C57BL6/J background. Rosa26-loxp-stop-loxp-RFP mice (male and female) were sacrificed at postnatal 0 day. APCmin/+ mice (male) were sacrificed at 10-week or 13-week.
Wild animals	The study did not involve in wild animals
Field-collected samples	The study did not involve in wild animals
Ethics oversight	All animal procedures were conducted in accordance with the National Institutes of Health Guide for the Care and Use of Laboratory Animals (NIH Publications No. 8023, revised 1978) and approved by the Animal Ethics Committee of Fudan University

Note that full information on the approval of the study protocol must also be provided in the manuscript.

Flow Cytometry

Plots

Confirm that:

- [x] The axis labels state the marker and fluorochrome used (e.g. CD4-FITC).
- [x] The axis scales are clearly visible. Include numbers along axes only for bottom left plot of group (a 'group' is an analysis of identical markers).
- [x] All plots are contour plots with outliers or pseudocolor plots.
- [x] A numerical value for number of cells or percentage (with statistics) is provided.

Methodology

Sample preparation	Mice were anesthetized with isoflurane, perfused with 4 °C phosphate buffer saline (PBS), and decapitated. Tumors were collected in 37℃ RPMI containing 1% FBS. Small pieces of tumors from mice were treated with 0.2 mg/ml collagenase (1 ml, 37 ° C, 15 min) supplemented with 0.1 mg/ml DNase I. Then, 1 ml of prewarmed 10% FBS was added to stop collagenase activity, followed by careful trituration. Blood was collected into anticoagulation tubes (BD), and red blood cells (RBCs) were removed by Red Blood Cell Lysis Buffer. Cell mixtures were passed through a sterile 70 µm filter and resuspended in 4 °C PBS 0.5% BSA solution for staining. The cells were incubated with primary antibody at 4 °C for 30 min. The FACS antibodies used are described above. Following washing with 300 µl PBS 0.5% BSA solution, the cells were centrifuged at 300 g for 3 min, and the supernatant was discarded. The secondary antibody was subjected to the same process for staining and washing as above. Finally, 300 µl PBS 0.15% BSA solution was added to resuspend the cells.
Instrument	BD FACSCabilur,BD FACSCanto II
Software	FlowJo (v7.6) software
Cell population abundance	at least 1000 cells after per group
Gating strategy	FSC: 200-800; SSC: 150-800; FITC/GFP/RFP/APC: >10^1

- [x] Tick this box to confirm that a figure exemplifying the gating strategy is provided in the Supplementary Information.
