# Supplementary figures and images for: Using apelin-based synthetic Notch receptors to detect angiogenesis and treat solid tumors
Source: Nat Commun. 2020 May 1;11:2163. doi: 10.1038/s41467-020-15729-4 (PMC7195494; doi:10.1038/s41467-020-15729-4)

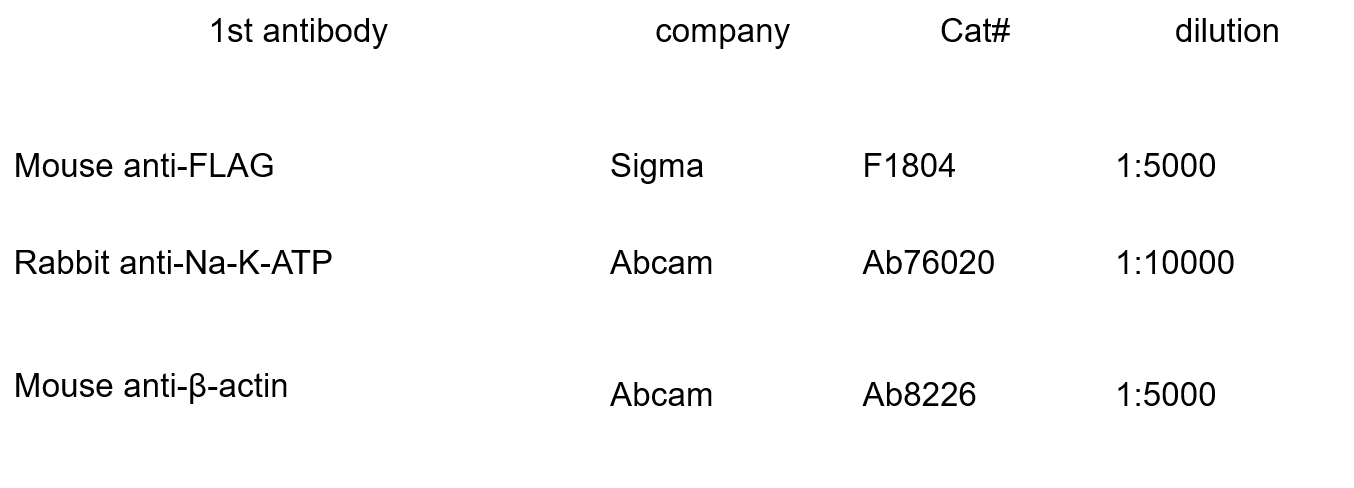
source data: western blots in manuscript

β-actin

Na-K-ATP

FLAG


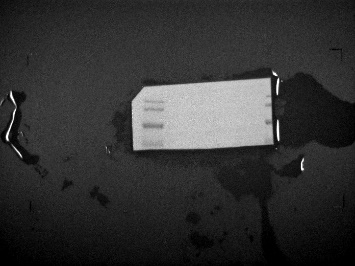

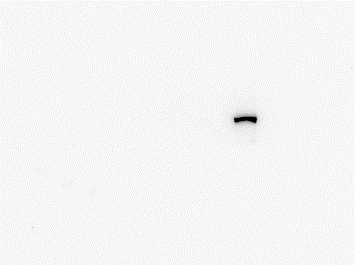


130 kDa

95 kDa


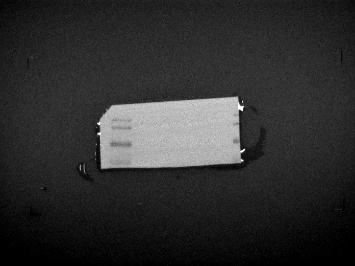

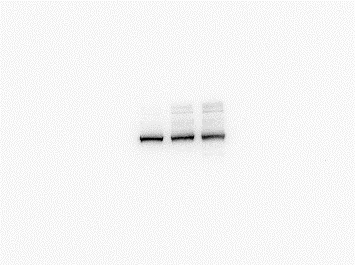


130 kDa

95 kDa


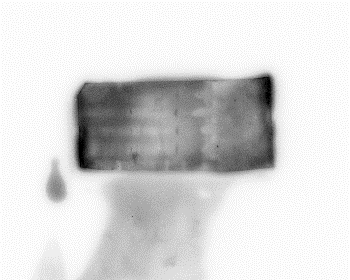

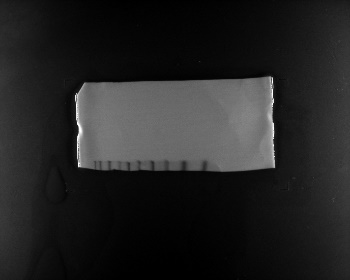


50 kDa

30 kDa

Supplement: Supplementary file 4 — Source Data [file 41467_2020_15729_MOESM4_ESM.docx]
